# Non-Surgical Locoregional Therapies Alone or in Combination with Systemic Therapy in Patients with Hepatocellular Carcinoma

**DOI:** 10.3390/cancers15061748

**Published:** 2023-03-14

**Authors:** Perla Chami, William Jarnagin, Ghassan K. Abou-Alfa, James Harding, Neal Kim, Haibo Lin, Maria El Homsi, Christopher Crane, Carla Hajj

**Affiliations:** 1 Department of Biology, Faculty of Arts and Sciences, American University of Beirut, Beirut 1107, Lebanon; 2 Memorial Sloan Kettering Cancer Center, New York, NY 10027, USA; 3 Department of Surgery, Weill Medical College at Cornell University, New York, NY 10021, USA; 4 Department of Medicine, Weill Medical College at Cornell University, New York, NY 10021, USA; 5 New York Proton Center, New York, NY 10035, USA

**Keywords:** hepatocellular carcinoma, combination therapy, ablation, interventional radiology, ablative radiation therapy, chemoembolization, radioembolization, proton beam therapy, stereotactic body radiation therapy

## Abstract

**Simple Summary:**

Ablation, arterially directed therapies and external beam radiation therapy are among the locoregional therapies considered for patients with hepatocellular carcinoma (HCC) who are ineligible for transplant or resection or as bridging therapy prior to transplant. Combinations of locoregional therapies along with systemic therapies constitute a growing area of interest with the objective of attaining superior therapeutic outcomes. We present herein an up-to-date review of the current and developing treatment modalities in this regard for HCC.

**Abstract:**

Hepatocellular carcinoma (HCC) is the most common primary liver cancer, representing the third-leading cause of cancer-related deaths worldwide. Curative intent treatment options for patients with HCC include liver transplantation, resection and ablation of small lesions. Other potentially curative therapies include cryoablation, microwave ablation and percutaneous alcohol injection. For locally advanced disease, different arterially directed therapies including transarterial chemoembolization and selective internal radiation therapy, plus external beam radiation including three-dimensional conformal radiation therapy, intensity-modulated radiation therapy, stereotactic body radiation therapy and proton beam therapy, are available or studied. Systemic therapies based on checkpoint inhibitors and tyrosine kinase inhibitors are available for the management of metastatic HCC and sometimes for locally advanced disease. Combinations of locoregional therapies with systemic drugs are currently the subject of several clinical trials.

## 1. Introduction

Liver cancer accounted for 8.3% of cancer-related mortality in 2020, making it the third-leading cause of cancer-related deaths globally [1]. Hepatocellular carcinoma (HCC) is the most commonly diagnosed primary liver malignancy. Its efficient management depends on early detection, the stage of progression of the disease and underlying liver function [2]. Cirrhosis, which can often result from infection by hepatitis B or C, overconsumption of alcohol or non-alcoholic steatohepatitis (NASH), continues to be the main risk factor leading to the development of HCC [3]. Available curative treatments include liver transplantation, resection and ablation. These are in addition to cryoablation, microwave ablation and percutaneous alcohol injection. In cases of the late detection of HCC, many patients with locally advanced disease and are offered locoregional therapies, including transarterial chemoembolization and selective internal radiation therapy, plus external beam radiation. In the case of advanced HCC, systemic therapies are the principal line of therapy, most notably immune checkpoint inhibitors and tyrosine kinase inhibitors [4]. 

In recent years, several studies have focused on evaluating the combination of locoregional treatments and systemic therapy. A few years ago, the only option patients with hepatocellular carcinoma had in terms of systemic therapy was sorafenib. This has changed dramatically with the recent emergence of new agents that are now proven to be effective in randomized trials, such as lenvatinib, durvalumab plus tremelimumab, atezolizumab plus bevacizumab, and single-agent durvalumab [5,6,7].

Studies of locoregional treatment options have also shown promising evidence supporting the use of ablative radiation therapy for non-transplant candidates in comparison with other local treatment techniques [8,9,10].

In this review, we present the current treatment modalities available as well as the latest advancements under investigation for the management of HCC.

## 2. Locoregional Therapies

**Figure 1:**
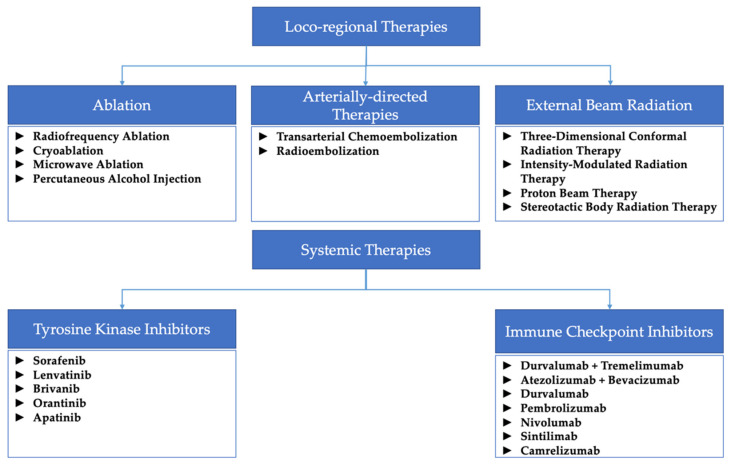
Diagram summarizing the locoregional and systemic therapies mentioned throughout this review.

Figure 1 summarizes all the different locoregional and systemic therapies mentioned throughout this paper.

### 2.1. Ablation 

#### 2.1.1. Radiofrequency Ablation

Radiofrequency ablation (RFA) is considered to be the preferred first-line locoregional treatment for patients with very early HCC, with single tumors <2 cm and who are not eligible for surgery [11,12]. Patients may be advised against surgery due to the tumor location, size or cirrhosis-related liver damage, which can restrict the amount of functional liver tissue that can be preserved [13]. RFA begins with the insertion of a single electrode into the target tissue with the aim of delivering a high-frequency alternating current (450–500 kHz). This process generates frictional heat at temperatures of 60–100 °C at the tip of the electrode, which is transmitted peripherally in all directions, leading to coagulative necrosis and tissue dehydration [14,15].

Stereotactic radiofrequency ablation (SRFA) involves the use of multiple radiofrequency probes under imaging guidance, which allows for 3D treatment planning and exact probe placement in combination with image fusion to track treatment progress [16]. In contrast to RFA, SRFA can be used to treat multiple and larger tumors during a single session, meaning patients with Barcelona Clinic Liver Cancer (BCLC) stage B and C are not advised against this procedure, as is the case with RFA [17]. A case–control study evaluating the efficacy and safety of SRFA in patients with subcardiac HCC reported complete tumor resolution after a single session in 95.6% of cases and a 7% local recurrence rate [17].

#### 2.1.2. Cryoablation

Cryoablation is an ablation method based on the cyclical exposure of target tumor sites to low temperatures, which can be performed using liquid nitrogen at −196 °C [18]. Owing to the high risk of complications with nitrogen, more recent technology has given way to the development of argon–helium cryotherapy. This method relies on cyclical freeze–thaw mechanisms, which involve the use of cryoprobes that are cooled using argon gas and then heated with helium gas [19]. As a result, tumor necrosis is induced through the formation of intracellular ice crystals, which cause damage to the integrity of the cell membranes and lead to the release of their contents into the extracellular space, thereby setting off the body’s immune defenses [20]. Among the advantages of cryoablation over RFA are its creation of greater ablation zones, the occurrence of less procedural pain and the development of ice balls generated by cryoprobes during the procedure, which allows intraprocedural monitoring [18,21]. A randomized control trial comparing treatment outcomes of percutaneous cryoablation with RFA found that the local progression rate in the cryoablation arm was significantly lower than in the RFA arm for lesions less than 3 cm in diameter (7.7% vs. 18.2%, respectively; *p* = 0.041). However, no significant differences were reported in terms of safety and efficacy between both methods [21]. A recently published systematic review showed no difference in overall survival or local progression of early and very early HCC between microwave ablation (MWA), RFA and cryoablation [22].

#### 2.1.3. Microwave Ablation

MWA is a minimally invasive thermal ablation technique used in the treatment of HCC. It involves the application of high frequency electromagnetic waves ≥900 MHz using needle-like microwave antennae that are inserted into the tumor site under ultrasound, CT or MRI guidance to induce tumor damage. MWA is preferably conducted percutaneously (P-MWA), which is the most minimally invasive of possible approaches, and can be repeated if tumor recurrence occurs. However, when tumors are not accessible percutaneously, MWA can also be performed laparoscopically (L-MWA), thoracoscopically or via laparotomy [23].

Numerous studies have attempted to compare MWA to RFA in terms of efficacy and safety. While both techniques generate high temperatures in target locations to coagulate tissue, they do so based on different mechanisms; RFA relies on electrical current, whereas MWA utilizes electromagnetic energy. Additionally, MWA possesses some advantages over RFA, including faster ablation time, the ability to deliver higher temperatures and the production of larger and more uniform ablation zones [23,24]. This is aided by the fact that MWA is less susceptible to the “heat sink” effect, which arises when tumors are located in the proximity of large blood vessels and are incompletely ablated due to heat loss [25]. In addition, there is no risk of burning, meaning it can be used in patients with metallic structures such as pacemakers who cannot be exposed to the electrical current of RFA [26]. Meta-analyses of P-MWA vs. percutaneous RFA (P-RFA) have reported no significant differences in complete ablation (CA) rates, local recurrence (LR) rates, disease free survival (DFS), overall survival (OS) and major complication rates between both methods. Moreover, no statistical differences were found in DFS, OS and CA rates between L-MWA and laparoscopic-RFA (L-RFA). However, L-MWA was shown to produce a significantly lower LR rate and a higher but non-significant major complication rate in comparison to L-RFA [24].

#### 2.1.4. Percutaneous Alcohol Injection

Percutaneous ethanol injection (PEI) is another treatment option for patients with early HCC who are not candidates for surgery. PEI is either performed in multiple sessions or in a single session under ultrasound guidance. Multiple sessions (3–12 sessions) are recommended for lesions measuring between 2 and 5 cm and are conducted without anesthesia. Small doses of ethanol are injected once or multiple times throughout a session, depending on the willingness of the patient, the length of the lesion and the ethanol distribution. The needle is left in place as the alcohol diffuses into the blood supply and is taken out once it begins to leak out of the lesion. Ethanol achieves tumor necrosis by causing instant cytoplasm dehydration and blood clotting, leading to ischemia. For patients with multifocal disease or lesions larger than 5 cm, a single-session treatment can be carried out with higher doses of ethanol under general anesthesia and by using endotracheal intubation with mechanical ventilation. Patients also receive a dose of fructose-1,6-diphosphate intravenously to accelerate ethanol’s metabolism and lessen its systemic effects [27].

A meta-analysis study comparing treatment with RFA vs. PEI by taking into account the results of five randomized controlled trials reported significantly higher OS and significantly lower recurrence risk in the RFA arm [28]. However, more recently, a randomized controlled trial comparing 5-year survival rates between both methods reported no statistically significant differences in OS (68% vs. 70% in the PEI and RFA groups, respectively; *p* = 0.451) [29]. Another meta-analysis conducted in 2022 comparing the efficacy of PEI vs. RFA in tumors smaller than 5 cm revealed that patients receiving treatment with RFA did not show significant increases in median OS or median cancer-specific survival in comparison to patients treated with PEI [30].

Table 1 summarizes the indications for locoregional therapies based on the BCLC stage, the size of the lesions and the presence of PVT.

### 2.2. Arterially Directed Therapies

Arterially directed therapies are preferred for patients with intermediate-stage HCC who are ineligible for transplant, resection or ablation. Intermediate-stage HCC is characterized by preserved liver function and the absence of vascular invasion or extrahepatic disease. Transarterial treatments make use of the dependence of hepatic tumors on blood supply delivered almost entirely by the hepatic artery [32]. This allows for the selective infusion of chemotherapeutic or radioactive agents exclusively to the tumor site with minimal consequences for the surrounding healthy liver tissue. Embolization of the blood vessel then blocks the incoming blood supply and leads to tumor necrosis.

#### 2.2.1. Transarterial Chemoembolization

The BCLC guidelines recommend transarterial chemoembolization (TACE) as the standard first-line treatment for intermediate HCC without vascular involvement due to its demonstration of consistent survival benefits in BCLC stage B patients [31]. TACE involves the administration of a cytotoxic drug, most often doxorubicin or cisplatin, emulsified in lipidol, a radio-opaque oil-based contrast agent. Intra-arterial injection is followed by embolization with embolic agents including gelatin sponge particles, polyvinyl alcohol, autologous blood clots and starch microspheres [33].

Subsequent technical adjustments gave rise to drug-eluting bead transarterial chemoembolization (DEB-TACE), which has the ability of administering embolic microspheres loaded with cytotoxic drugs into the hepatic artery in a more sustained and controlled fashion. This enables the maintenance of high concentrations of the drug at the tumor location for a period lasting from several days to one month.

Multiple randomized control trials have attempted to compare TACE and DEB-TACE in terms of OS, safety and efficacy but have failed to report any significant differences. The PRECISION V multicenter randomized phase II trial aimed to evaluate differences between TACE and DEB-TACE, with safety and tumor response rate 6 months post-treatment as primary endpoints. The objective response (OR) rate at 6 months was reported to be 51.6% vs. 43.5% in the DEB-TACE vs. TACE groups, respectively (*p* = 0.11). Safety, defined as the incidence of treatment-related serious adverse events within 30 days of the procedure, was found to be 20.4% vs. 19.4% in the DEB-TACE vs. TACE groups, respectively (*p* = 0.86). Therefore, it was concluded that DEB-TACE could not demonstrate statistical superiority over TACE in terms of treatment efficacy and safety [34]. Similarly, the PRECISION ITALIA phase III trial failed to show significant differences in survival, tumor response, time to progression (TTP) and safety between both procedures. However, DEB-TACE was linked to less post-procedural abdominal pain in comparison with TACE [35,36]. Moreover, retrospective studies comparing both treatments have shown that DEB-TACE was associated with a significantly higher incidence of hepatic artery damage following a single session (OR, 6.36, *p* <0.001), as well as a more frequent risk of presenting with at least one liver or biliary injury in cirrhotic HCC patients (30.4% in DEB-TACE vs. 4.2% in TACE; *p* <0.001) [37].

Despite the popularity of TACE, the mode of action of chemotherapeutic agents remains unclear. A randomized phase 2 trial comparing treatment of HCC with TACE microspheres loaded with doxorubicin versus transarterial embolization (TAE) with microspheres alone revealed no significant differences in response rate, progression-free survival (PFS) and OS, suggesting that the efficacy of TACE may be mainly due to ischemic action [38]. Similarly, a recent study comparing initial response rates in HCC patients that underwent treatment with either TAE or DEB-TACE found no significant differences between both arms [39].

#### 2.2.2. Radioembolization

Radioembolization (RE) or selective internal radiation therapy (SIRT) is another type of arterially targeted locoregional therapy, which involves the selective infusion of radioactive microspheres, serving as carriers of the β-emitter yttrium-90 (Y-90), through the hepatic artery. The high-energy β-radiation emitted is capable of creating breaks in the double-stranded DNA, thereby causing damage to the tumor cells [35]. The small dimensions of the microspheres and their poor ability to penetrate into tissues, and Y-90′s half-life of 2.67 days, allow for minimal exposure of the surrounding liver parenchyma and increased tumor targeting [35,40]. Two types of Y-90 microspheres are currently available commercially: resin and glass microspheres. They vary in size, dosimetry, radioactivity per microsphere and the number of microsphere required to be injected [35].

SIRT with Y-90 is typically performed as first-line treatment in patients with intermediate HCC that are ineligible for TACE or treatment with systemic therapy [35]. It can be used in patients with advanced HCC with portal vein involvement [15]. Promising results were demonstrated in a cohort study evaluating OS following SIRT with Y-90 in HCC patients with different stages of disease progression (24.4, 16.9 and 10.0 months for early, intermediate and advanced HCC, respectively) [41]. Several retrospective and prospective studies sought to compare the efficacy of TACE vs. RE with Y-90 and revealed no significant differences in OS between both procedures, even among different stages of HCC [42]. In addition, multiple randomized controlled trials found no significant improvement of OS in SIRT with Y-90 over sorafenib (Table 2) [40,43,44]. Results from the SARAH trial in France showed an OS of 8.0 vs. 9.9 months in the SIRT vs. sorafenib arms, respectively (*p* = 0.18) [40]. Similarly, the SIRveNIB phase 3 trial in Asia/Pacific patients reported an OS of 8.8 vs. 10.0 months in the SIRT vs. sorafenib groups, respectively (*p* = 0.36) [43]. The combination of SIRT with FOLFOX chemotherapy over FOLFOX alone in patients with liver metastases from colorectal cancer in the FOXFIRE, SIRFLOX and FOXFIRE-Global trials also resulted in no significant differences in OS between both treatment arms (22.6 vs. 23.3 months in SIRT + FOLFOX vs. FOLFOX alone, respectively; *p* = 0.61; Table 2) [45]. The negative outcomes of these trials could be due to Y-90′s small mean penetration depth of 2.5 mm and its characteristic heterogeneous dose distribution at the peripheries of tumors rather than the center, resulting in treatment being limited by the anatomy of the vessels surrounding the tumor nodules [35,46]. The doses administered are also not under the radiology oncologist’s control, but rather determined empirically according to published data or based on the body surface area method [35]. RE with Y-90 does exhibit potential toxic effects because of unintended high-intensity irradiation to non-target organs, which can cause the development of radiation-induced liver disease (RILD). Additionally, patients with bilirubin levels greater than 2 mg/dL present with a higher risk of developing RILD after RE with Y-90 treatment [4].

Table 2 includes a series of randomized controlled trials evaluating treatment outcomes with Y-90.

### 2.3. External Beam Radiation Therapy

#### 2.3.1. Three-Dimensional Conformal Radiation Therapy

At the molecular level, radiation is capable of inducing DNA damage in cells, which results in their inability to proliferate [47]. Three-dimensional conformal radiation therapy (3DCRT) is a type of high-energy photon radiotherapy, which makes use of CT imaging to identify the gross tumor target in the liver and subsequently design the arrangement of multiple radiation beams to deliver the dose to the planning target volume (PTV) while protecting surrounding organs at risk [2]. Treatment planning with CT imaging is performed with the patient in a supine position while immobilized in a mold, with both arms abducted and externally rotated above the head [48,49]. Patients can also be trained to hold their breath during the inspiratory or expiratory phase to limit the movement of the target site [50]. The volumes and structures contoured include gross tumor volume (GTV), clinical target volume (CTV), PTV and organs at risk (OARs) [48]. Then, radiation is delivered with multiple photon beams of up to 15 MV, with the possible addition of 1–2 non-coplanar beams to enhance the conformality of radiation treatment [48,50]. Among the advantages of 3DCRT is its ability to precisely deliver elevated doses of radiation regardless of tumor size, topography or presence of segmental portal vein thrombosis, while sparing the surrounding healthy liver parenchyma and nearby organs. Moreover, 3DCRT’s efficacy is strongly associated with elevated radiation doses and smaller nodule sizes [51]. A study evaluating whether higher fraction doses with 3DCRT resulted in survival benefits for patients with HCC tumors less than 10 cm found that median survival was 14.4 vs. 24.8 months (*p* = 0.003) in the groups receiving 2.5–4.9 Gy vs. 5.0–7.0 Gy three times a week, respectively. This survival benefit was observed without any additional toxicities in the group receiving the higher fraction dose of treatment [52].

#### 2.3.2. Intensity-Modulated Radiation Therapy

Advances in radiotherapy techniques gave rise to intensity-modulated radiation therapy (IMRT), which has the ability to distribute higher doses of radiation to more complex target shapes compared to 3DCRT while limiting exposure to the surrounding tissue [53]. As opposed to 3DCRT’s feature of using fields conforming to the outline of the tumor site to direct a homogenous intensity of radiation, IMRT splits the conformal fields into multiple subfields to deliver a non-uniform intensity distribution. This allows for better control and precision as radiation-resistant areas can be irradiated with higher doses while sensitive areas can receive conventional or reduced doses, as it achieved by using dose-painting techniques or simultaneous integrated boosts [53,54,55]. In order to create an intensity-modulated field, a conventional multileaf collimator (MLC) delivers radiation in one of three possible modes: (1) step-and-shoot, (2) sliding window and (3) volumetric modulated arc therapy (VMAT). In static IMRT, the MLC leaves only move to generate the next dose segment when the radiation beam is switched off. In sliding window IMRT, the MLC leaves move while radiation is being distributed, and the beam intensity is varied in multiple static fields through modulated MLC velocity. VMAT is a rotational form of IMRT involving changing dose rates and gantry speeds while the MLC leaves are moving with a rotating gantry [56,57]. Comparisons between static IMRT and VMAT have demonstrated greater dose conformity, lower doses to OARs and reduced treatment times for VMAT [58]. Another IMRT technique is helical IMRT (h-IMRT), which requires an independent rotational helical tomotherapy machine equipped with a binary MLC and a megavoltage CT imaging system in order to deliver radiation doses through a rapidly rotating X-ray source in a CT-like gantry [54,56]. A retrospective study comparing h-IMRT to 3DCRT showed significant differences in the local control (LC) rate (46.8% for h-IMRT and 28.2% for 3DCRT; *p* = 0.007) and 3-year OS (33.4% for h-IMRT and 13.5% for 3DCRT; *p* <0.001). No significant differences were found for RILD between both methods (*p* = 0.716) [59].

#### 2.3.3. Proton Beam Therapy

While the use of photons in radiotherapy has been shown to increase dose conformity and allow for dose escalation, a considerable portion of surrounding liver parenchyma is exposed to radiation, which puts cirrhotic HCC patients at risk of developing RILD and possibly liver failure. The increased dose to the normal liver parenchyma in 3DCRT and IMRT planning is mainly due to the exit dose of high-energy X-rays on the beam path. In contrast, protons have a finite range for dose deposition and exhibit minimal energy loss along the length of a beam. Any residual energy lost close to the end of that range occurs over a very small distance. The outcome observed is called a “Bragg peak”, which is a sharp rise in the absorbed dose by the target tissue, resulting in little-to-no exit dose [60]. As with conventional RT, proton beam therapy (PBT) delivered at high ablative doses with fractionation techniques improved LC and OS rates in patients with bilobar colorectal metastases, HCC and ICC (Table 3) [61,62]. Research comparing PBT to other therapies for treating HCC has shown promising results. A randomized clinical trial comparing treatments with PBT vs. TACE showed similar OS rates but significant differences in 2-year LC (88% with PBT vs. 45% with TACE; *p* = 0.06) and PFS (48% for PBT vs. 31% for TACE; *p* = 0.06). The study also reported significantly fewer hospitalization days following PBT treatment [63]. Another randomized phase III trial concluded the non-inferiority of treatment with PBT when compared to RFA after finding a 2-year local PFS rate of 92.8% with PBT vs. 83.2% with RFA (*p* <0.001) [64]. Comparisons between PBT, VMAT and h-IMRT have also shown significantly better sparing of the liver and OARs with PBT [65]. A randomized trial comparing survival outcomes between proton and photon therapy in patients with unresectable HCC concluded there was improved OS (31 vs. 14 months; *p* = 0.008) in treatment with PBT compared to treatment with IMRT/VMAT, respectively (Table 3) [66]. Currently, an ongoing phase 3 trial (NCT03186898) is looking to compare OS as a primary endpoint in HCC patients treated with proton vs. photon radiation therapy (Table 3).

#### 2.3.4. Stereotactic Body Radiation Therapy

Stereotactic body radiation therapy (SBRT) is an advanced method of hypofractionated external beam radiation therapy (EBRT), which makes use of photons or protons to deliver high ablative radiation doses. It can act as an alternative to ablation and embolization therapies or can be opted for when these options are contraindicated or unsuccessful. SBRT is commonly performed in patients with 1–3 tumors with non-existent or minimal extrahepatic disease, but it can also be presented to patients with larger lesions or advanced disease as long as there remains an adequate amount of functional liver tissue and radiation is delivered within the accepted tolerated doses [4]. The delivery of high-dose radiation in SBRT is achieved through several modulated coplanar or non-coplanar beams or radiation arcs, which lead to the generation of a “hotspot” within the target site. This region receives a considerable amount of radiation while surrounding tissue is exposed to lower doses owing to a rapid dose fall-off gradient past the target [2,67]. To limit tumor motion throughout the procedure, physicians can apply motion management techniques, including breath-holding techniques, abdominal compression or respiratory gating [68]. Delineating the full extent of the tumor for treatment planning of SBRT can be achieved using a 4D CT, with time being the fourth dimension, which permits the visualization of the tumor throughout a breathing cycle, as is used to determine the internal target volume (ITV). MRI or CT angiography of the liver can also be applied and registered (fused) to the treatment planning CT scan, whenever feasible, for precise delineation of the tumor [2,69]. Additionally, gold fiducial markers can be implanted in the periphery of the lesions under image guidance and while the treatment is actively being delivered, certain LINACs have the capability of triggering imaging, producing a kV image at intervals of elapsed time, MU delivery, gantry angle or breathing motion of the patient to track tumor motion in real time [70,71]. The advantages of SBRT over IMRT are its ability to deliver higher radiation doses per treatment and its shorter overall treatment time [72]. Radiation treatment with either SBRT or fractionated RT can be ablative at a higher biologically effective dose (BED) (≥80 Gy), and was shown to improve hepatic tumor control, and possibly improve overall survival by preventing liver failure originating from tumor progression, when compared with the delivery of a lower BED in HCC patients ineligible for curative therapies (Table 3) [73]. In patients with intrahepatic cholangiocarcinoma (ICC), a higher BED (>80.5 Gy), when compared to lower doses, was associated with significantly better 3-year OS (73% vs. 38%, respectively; *p* = 0.17) and 3-year LC (78% vs. 45%, respectively; *p* = 0.04; Table 3) [74]. Fractionation methods can be used to perform ablative RT with increased radiation doses (90–100 Gy BED) and achieve improved survival in patients with larger liver nodules [75]. A recent retrospective study comparing treatment with SBRT to IMRT in 287 HCC patients with portal vein thrombosis found that there were no significant differences in OS, LC, PFS and intrahepatic control between both techniques. SBRT and IMRT were delivered in 3–5 vs. 7–20 fractions, respectively, with a final median radiation dose of 42 Gy vs. 51 Gy, respectively [76].

Currently, an ongoing prospective clinical trial (NCT04682847) on the use of SBRT for patients with primary and metastatic hepatic malignancies is looking to assess treatment response following the use of super paramagnetic iron oxide nanoparticles (SPION) during treatment planning together with a linear accelerator integrated to an MRI scanner (MR-Linac). Ferumoxytol, a SPION coated with semi-synthetic carbohydrates of low molecular weight, is able to remain within hepatic Kupffer cells and thus allow visualization of target sites on MR-Linac. Moreover, the use of SPIONs is also associated with their ability to protect human monocytes from radiation-induced cell death and cell cycle arrest [77]. Increasing evidence is pointing to the effectiveness of using SBRT as bridging therapy. A retrospective study looking to assess the effectiveness of SBRT in patients with HCC before undergoing transplant found that of the 35 out of 38 patients who were initially ineligible for liver transplantation, all ended up receiving transplants, with a 5-year OS of 73% [78].

Table 3 includes the outcomes of a series of studies assessing treatment with different modes of radiation therapy.

**Table 3:** Outcomes of selected studies on treatment with radiation therapy.

Study	Treatment Arms	Primary Endpoints	Outcomes	*p*-Value
Hong et al. [62]	PBT (n = 83)	LC (2 years)	94.8% (HCC); 94.1% (ICC)	NA
		OS (2 years)	63.2% (HCC); 46.5% (ICC)	
Sanford et al. [66]	PBT (n = 49) vs. IMRT/VMAT (n = 84)	OS (median)	31 vs. 14 months	*p* = 0.008
Tao et al. [74]	Ablative RT (n = 19) vs. RT (n = 60)	OS (3 years)	73% vs. 38%	*p* = 0.017
		LC (3 years)	78% vs. 45%	*p* = 0.04
Hilal et al. [73]	Ablative RT (n = 14) vs. non-ablative RT (n = 31)	LC (1 year)	91.7% vs. 75.2%	*p* = 0.038
		OS (median)	28 vs. 7 months	*p* = 0.044
NRG-GI003 NCT03186898 ^a^	Photon RT vs. proton RT (n = 186)	OS	Ongoing	

PBT: proton beam therapy; IMRT: intensity-modulated radiation therapy; VMAT: volumetric modulated arc therapy; RT: radiation therapy; SBRT: stereotactic body radiation therapy; LC: local control; OS: overall survival; HCC: hepatocellular carcinoma; ICC: intrahepatic cholangiocarcinoma; ASTRO: American Society for Radiation Oncology; ^a^ ongoing recruiting trial.

## 3. Combination Therapies

Combination therapies constitute a growing area of study for the treatment of HCC, with the aim of improving OS and achieving superior therapeutic outcomes. Table 4 highlights the outcomes of several randomized controlled trials on locoregional and systemic therapy in combination or alone, including ongoing combination trials. Historically, the multi-tyrosine-kinase inhibitor sorafenib was the recommended mode of systemic treatment in patients who underwent unsuccessful locoregional therapies or those with more advanced HCC [4,11,12]. Tyrosine kinase inhibitors function by targeting pro-angiogenic molecules in the microenvironment of a tumor, which leads to the inhibition of angiogenesis and cell proliferation, resulting in cell apoptosis [79]. Immune checkpoint inhibitors constitute another form of systemic therapy, in which they target immune cells to block the inhibitory action of immune checkpoint proteins that are dysregulated in cancer cells. An example of such a protein is the inhibitory T-cell receptor programmed cell death protein 1 (PD-1), which when blocked, can trigger antitumor immune responses [80]. Recent advances showed improved survival with immune checkpoint-inhibitor-based combination therapies including atezolizumab plus bevacizumab, and durvalumab plus tremelimumab, over sorafenib as first-line therapy in advanced HCC (Table 4) [5,7]. This is added to the use of single-agent sorafenib, lenvatinib or durvalumab [31]. The combination of lenvatinib plus pembrolizumab compared to lenvatinib alone did not achieve pre-specified statistical significance in median OS (21.2 vs. 19.0 months; *p* = 0.0227) and PFS (8.2 vs. 8.0 months; *p* = 0.0466) for the combination vs. monotherapy groups, respectively (Table 4) [81].

Multiple trials have attempted to assess the efficacy and safety of arterially directed therapies combined with systemic therapies for the treatment of HCC. A combination of DEB-TACE with sorafenib vs. DEB-TACE alone in the phase 2 SPACE and phase 3 TACE 2 randomized controlled trials failed to display improved TTP and PFS, respectively, in the combination group (Table 4) [82,83]. TACE has also been combined with other antiangiogenic drugs, in the BRISK-TA trial with brivanib and in the ORIENTAL trial with orantinib. Both studies failed to show better OS in the combination group (Table 4) [84,85]. However, a phase II RCT (TACTICS) combining TACE with sorafenib reported significantly improved PFS (25.2 months in combination group vs. 13.5 months in TACE; *p* = 0.006; Table 4) and time to untreatable (unTACEable) progression (26.7 months in combination group vs. 20.6 months in TACE; *p* = 0.02) [86]. SIRT with Y-90 has also been combined with sorafenib and compared to treatment with sorafenib alone in a phase 2 multicenter SORAMIC trial. The study reported no significant improvement of OS with combination therapy in the intention-to-treatment cohort (12.1 vs. 11.4 months in combination and sorafenib0only groups, respectively; *p*= 0.9529) or in the per-protocol (PP) population (14.0 vs. 11.1 months in combination and sorafenib-only groups, respectively; *p*= 0.2515). However, subgroup analyses of the PP population have shown improved survival in patients without cirrhosis, cirrhosis of non-alcohol etiology and patients ≤65 years old [44]. Moreover, trials combining checkpoint inhibitors with locoregional therapies have also been conducted. In one study, the feasibility of tremelimumab combined with RFA, cryoablation or TACE was demonstrated, and treatment led to an increase in intratumoral CD8^+^ T-cells. However, the role of ablation or TACE in amplifying the effects of tremelimumab remains unclear [87]. More recently, an ongoing phase 1 trial (NCT03143270) combining nivolumab with DEB-TACE also reported safety of treatment in patients with unresectable HCC [88].

In a Cochrane review, it was noted that no confirmed randomized clinical trials have been conducted in patients with HCC to assess the beneficial or harmful effects of TACE plus ablation versus TACE alone [89].

Studies on the combination of TACE plus radiation therapy have shown more promising results. A study assessing survival and tumor response in ≤5 cm HCC patients undergoing SBRT with TACE vs. TACE alone found significantly greater 1- and 3-year LC rates in the combination group (91.1% and 89.9%, respectively; *p* <0.001) vs. the TACE group (69.9% and 44.8%, respectively; *p* <0.001) and significantly better 1- and 3-year PFS in the combination group (56.5% and 32.3%, respectively; *p* = 0.022) vs. the TACE group (42.2% and 21.6%, respectively; *p* = 0.022). However, combination therapy was associated with improved PFS only in patients with ≤2 tumors. No significant differences were found in OS between both groups [90]. Another retrospective study looking to assess survival in patients with >5 cm HCC undergoing treatment with SBRT followed by TACE vs. SBRT monotherapy found significantly higher median OS in the combination group compared to the SBRT group (42.0 vs. 21.0 months, respectively; *p* = 0.047) [91]. Moreover, a recent retrospective study compared OS and PFS between two combination treatments: TACE with SBRT vs. TACE with sorafenib. Significant differences were found in median OS (24.2 vs. 12.9 months; *p* = 0.001) and median PFS (10.1 vs. 3.6 months; *p* < 0.001) in the TACE-SBRT vs. TACE-sorafenib groups, respectively [92].

Randomized phase 3 trial RTOG-1112 (NCT01730937) aimed to assess OS, PFS, TTP and toxicity in patients with advanced HCC undergoing treatment with SBRT followed by sorafenib vs. sorafenib alone. The results showed an increase in the primary endpoint, median OS, in the combination arm over the monotherapy arm (15.8 vs. 12.3 months, respectively; *p* = 0.0554; Table 4). Similarly, median PFS was improved with SBRT + sorafenib vs. sorafenib alone (9.2 vs. 5.5 months, respectively; *p* = 0.0001), as well as TTP (18.5 vs. 9.5 months, respectively; *p* = 0.034) [93,94].

In a recent case series, the possible combination of TACE, MWA, tyrosine kinase inhibitor apatinib and immunotherapy with camrezilumab was suggested to treat patients with advanced HCC. However, the safety and efficacy of this treatment method have not been the subject of any clinical trials to date [95].

**Table 4:** Outcomes of select randomized controlled trials on locoregional and systemic therapies.

Randomized Controlled Trial	Treatment Arms	Primary Endpoints	Outcomes	*p*-Value
Locoregional therapies				
PRECISION V [34]	TACE (n = 108) vs. DEB-TACE (n = 93)	Tumor response at 6 months	22.2% vs. 26.9%	*p* = 0.11
		Treatment-related adverse events within 30 days of a procedure	19.4% vs. 20.4%	*p* = 0.86
PRECISION ITALIA [96]	TACE (n = 88) vs. DEB-TACE (n = 89)	OS (2 years)	55.4% vs. 56.8%	*p* = 0.949
Systemic therapies alone				
IMbrave150 [5]	Atezolizumab + bevacizumab (n = 336) vs. sorafenib (n = 165)	OS (1 year)	67.2% vs. 54.6%	*p* < 0.001
		PFS (median)	6.8 vs. 4.3 months	*p* < 0.001
HIMALAYA [7]	Tremelimumab + durvalumab (n = 393) vs. sorafenib (n = 389)	OS (median)	16.4 vs. 13.8 months	*p* = 0.0035
	Durvalumab (n = 389) vs. sorafenib (n = 389) noninferiority	OS (median)	16.6 vs. 13.8 months	*p* = 0.0035
LEAP-002 ^c^ [81]	Lenvatinib + pembrolizumab vs. lenvatinib alone	OS (median)	21.2 vs. 19.0 months	*p* = 0.0227
		PFS (median)	8.2 vs. 8.0 months	*p* = 0.0466
Systemic therapies in combination with locoregional therapies				
SPACE [82]	DEB-TACE + sorafenib (n = 154) vs. DEB-TACE + placebo (n = 153)	Time to tumor progression	169 vs. 166 days	*p* = 0.072
TACE 2 [83]	DEB-TACE + sorafenib (n = 157) vs. DEB-TACE + placebo (n = 156)	PFS (median)	238.0 vs. 235.0 days	*p* = 0.94
BRISK-TA [84]	TACE + brivanib (n = 249) vs. TACE + placebo (n = 253)	OS (median)	26.4 vs. 26.1 months	*p* = 0.5280
ORIENTAL [85]	TACE+ orantinib (n = 444) vs. TACE + placebo (n = 444)	OS (median)	31.1 vs. 32.3 months	*p* = 0.435
TACTICS [86]	TACE+ sorafenib (n = 80) vs. TACE alone (n = 76)	PFS (median)	25.2 vs. 13.5 months	*p* = 0.006
RTOG-1112 [93] NCT01730937	Sorafenib + SBRT (n = 85) vs. sorafenib alone (n = 92)	OS (median)	15.8 vs. 12.3 months	*p* = 0.0554
NCT04857684 ^a^	Atezolizumab + bevacizumab + SBRT (n = 20)	Proportion of patients with grade 3–4 treatment-related adverse events		
NCT05096715 ^b^	Atezolizumab + bevacizumab + SBRT (n = 20)	Dose-limiting toxicity rate		
NCT05377034 ^b^	Atezolizumab + bevacizumab + SIRT-Y-90 (n = 88) vs. SIRT-Y-90 + placebo (n = 88)	BORR		
NCT04541173 ^a^	Atezolizumab + bevacizumab + TARE-Y-90 vs. TARE-Y-90 alone (n = 128)	PFS		
NCT04712643 ^a^	Atezolizumab + bevacizumab + TACE vs. TACE alone (n = 342)	TACE PFS OS		
NCT04913480 ^a^	Durvalumab + SBRT (n = 37)	PFS (1 year)		
NCT04167293 ^a^	Sintilimab (n = 58) + SBRT vs. SBRT alone (n = 58)	PFS (24 weeks)		
NCT04387695 ^a^	Sorafenib + SBRT + TACE vs. sorafenib alone (n= 54)	PFS (12 weeks)		
EMERALD-3 NCT05301842 ^a^	Durvalumab + tremelimumab + TACE +/− lenvatinib vs. TACE alone (n = 525)	PFS		
LEAP-012 NCT04246177 ^a^	Lenvatinib + pembrolizumab + TACE vs. TACE + placebo + placebo (n = 950)	PFS OS		
NCT05286320 ^a^	Lenvatinib + pembrolizumab + SBRT (n = 27)	Safety rate ORR		

TACE: transarterial chemoembolization; DEB-TACE: drug-eluting bead TACE; SBRT: stereotactic body radiation therapy; SIRT: selective internal radiation therapy; Y-90: yttrium-90; TARE: transarterial radioembolization; OS: overall survival; PFS: progression-free survival; BORR: best overall response rate; ORR: objective response rate; ^a^ ongoing recruiting trial; ^b^ not yet recruiting trial; ^c^ primary results.

## 4. Ongoing Clinical Trials

There are currently multiple ongoing trials looking to study the efficacy and safety of radiotherapy and/or embolization in combination with systemic therapies (Table 4). The rationale behind the combination of radiation therapy and immune checkpoint inhibitors stems from the ability of radiotherapy to recruit T-cells to the tumor site, as well as vascular adhesion molecules that aid in the infiltration of T-cells through the release of chemokines, which creates an active immune microenvironment for the immune checkpoint inhibitors to act on [97]. Following the favorable outcomes of the IMbrave150 trial, atezolizumab combined with bevacizumab is being subject to further research in combination with SBRT (e.g., NCT04857684, NCT05096715), Y-90 (e.g., NCT05377034, NCT04541173) and TACE (NCT04712643). SBRT is also being studied in combination with different systemic therapies in currently recruiting trials, such as durvalumab (NCT04913480) and sintilimab (NCT04167293). Another phase 3 randomized controlled trial in China is currently recruiting HCC patients with BCLC stage C and portal vein tumor thrombus to either undergo treatment with SBRT + TACE + sorafenib or sorafenib alone with the aim of assessing PFS between both groups (NCT04387695). Moreover, a multicenter phase 3 trial (EMERALD-3) is looking to compare combined treatment of durvalumab + tremelimumab + TACE with or without lenvatinib in comparison with TACE alone in locoregional HCC (NCT05301842). Another multicenter randomized phase 3 trial (LEAP-012) aims to compare OS and PFS between intermediate-stage HCC patients undergoing treatment with TACE combined with lenvatinib and pembrolizumab vs. TACE alone (NCT04246177). Similarly, a phase Ib/II trial is set to assess the safety and objective response rate in patients with portal vein thrombosis when combining lenvatinib and pembrolizumab with SBRT (NCT05286320).

## 5. Conclusions

Sequential treatment using locoregional therapies (ablation, arterially directed therapies, external beam radiation therapy) and systemic therapies (sorafenib, durvalumab, lenvatinib, atezolizumab/bevacizumab) remains the standard of care for patients with unresectable HCC. However, the optimal timing of combining locoregional and systemic therapies remains unknown. Results from the SORAMIC, SPACE, TACE-2, ORIENTAL and BRISK-TA trials all failed to demonstrate improved survival with the combination of arterially directed therapies (Y-90, DEB-TACE and TACE) and systemic drugs [44,82,83,84,85]. However, improved PFS was reported in the TACTICS trial combining TACE with sorafenib [86]. Recent results from the RTOG-1112 trial at the ASTRO 2022 meeting also demonstrated improved OS, PFS and TTP when combining SBRT followed by sorafenib vs. sorafenib alone. Promising findings on ablative radiation therapy could mean significantly improved outcomes for patients with unresectable HCC [73,74,75]. Currently, several ongoing clinical trials intend to assess the outcomes of combining systemic therapy with radiation therapy (e.g., NCT04857684, NCT04913480, NCT04167293, NCT05286320).

## Figures and Tables

**Table 1:** Indications for locoregional therapies.

	BCLC Stage	Size [31]	Location Contraindications [15]	Presence of PVT [15]
Ablation	A	≤3 cm	Proximity to major bile ducts, large vessels, intra-abdominal organs and subcapsular regions	No
Arterially directed therapies	B	≤8 cm (for RE)	Extensive bilobar involvement	No (TACE) Yes (RE)
Radiation	A or B	no limitation	Extrahepatic disease	Yes

BCLC: Barcelona Clinic Liver Cancer; PVT: portal vein thrombosis; TACE: transarterial chemoembolization; RE: radioembolization.

**Table 2:** Outcomes of selected randomized controlled trials for treatment with Y-90.

Randomized Controlled Trial	Treatment Arms	Primary Endpoint	Outcomes	*p*-Value
SARAH [40]	SIRT-Y-90 (n = 237) vs. sorafenib (n = 222)	OS	8.0 vs. 9.9 months	*p* = 0.18
SIRveNIB [43]	SIRT-Y-90 (n = 182) vs. sorafenib (n = 178)	OS	8.8 vs. 10.0 months	*p* = 0.36
FOXFIRE, SIRFLOX, FOXFIRE-Global [45]	SIRT-Y-90 + FOLFOX (n = 554) vs. FOLFOX alone (n = 549)	OS	22.6 vs. 23.3 months	*p* = 0.61
SORAMIC [44]	SIRT-Y-90 + sorafenib (n = 216) vs. sorafenib (n = 208)	OS	12.1 vs. 11.4 months	*p* = 0.9529

SIRT: selective internal radiation therapy; Y-90: yttrium-90; OS: overall survival.
